# Platinum-Catalyzed Hydrative Cyclization of 1,6-Diynes for the Synthesis of 3,5-Substituted Conjugated Cyclohexenones

**DOI:** 10.3390/molecules15075045

**Published:** 2010-07-23

**Authors:** Chen Zhang, Jian-Feng Qi, Dong-Mei Cui, Qian Wang, Xiu-Li Wang

**Affiliations:** 1 School of Pharmaceutical Sciences, Zhejiang University, Hangzhou 310058, China; 2 College of Pharmaceutical Science, Zhejiang University of Technology, Hangzhou 310014, China; 3 College of Chemistry and Bioengineering, Guilin University of Technology, Guangxi, Guilin, China

**Keywords:** diyne, catalysis, hydrative cyclization, cyclohexenones

## Abstract

We have developed a Pt(COD)Cl_2_-catalyzed hydrative cyclization of 1,6-diynes leading to the formation of functionalized cyclohexenones in good yields.

## 1. Introduction

Cyclohexenone derivatives are not only key intermediates in organic synthesis, but they also exhibit important pharmacological activities [1,2,3,4]. Extensive synthetic efforts for conjugate cyclohexenones have been reported, in which an annulation approach from acyclic precursors constitutes a useful entry [5,6,7,8,9,10,11,12,13]. Despite this advance, there is still a great need to develop more convenient catalytic systems that can accommodate such attractive features as easily accessible starting materials, mild reaction conditions, and absence of co-products. The newly developed metal-catalyzed hydrative cyclization reaction is not only an especially attractive “green” procedure, but also an ideal synthetic method for preparing cyclic enone compounds [14,15,16,17,18,19,20]. The reported examples include hydrative cyclization of 1,n-diynes [14,15,16,17], 1-yne-5-enones [18], 1-en-5-ynes [19] and diynnols [20]. Recently, Liu and co-workers reported a PtCl_2_-catalyzed hydrative cyclization of internal triynes to yield bicyclic spiroketones [21,22,23]. As part of our ongoing studies on metal-catalyzed atom-economical reactions, we succeeded in synthesizing conjugate cyclohexenone ring systems using the hydrative cyclization of 1,6-diynes with (PPh_3_)AuMe as a catalyst [24,25]. Herein, we report our studies on the use of Pt(COD)Cl_2_ as a catalyst in this cyclization.

## 2. Results and Discussion

Initial hydrative cyclization experiments of 1,6-diyne **1a** (0.5 mmol) with H_2_O (0.5 mmol) at 70 °C for 4 h in a sealed-tube were performed to screen catalysts. Pt(COD)Cl_2_ (COD = cyclooctadiene) combined with methanesulfonic acid (CH_3_SO_3_H) showed good catalytic activity in this reaction, furnishing cyclohexenone **2a** in 75% yield without the formation of the corresponding hydration or methanol adducts (Table 1, entry 1), while the reaction conducted in the absence of CH_3_SO_3_H did not yield the cyclic product (Table 1, entry 2). Trifluromethanesulfonic acid (CF_3_SO_3_H) can also serve as an excellent co-catalyst. PtCl_2_ in combination with PPh_3_ gave **2a** in lower yield (Table 1, entry 4). There was no reaction with other homogeneous metal complex systems, such as Pd(P*^i^*Pr_3_)_2_Cl_2_ and Ru(COD)Cl_2_ (Table 1, entries 5 and 6). During further optimization of the reaction conditions, we found that a lower catalyst loading (2 mol%) afforded the product with decreased yield (Table 1, entry 3).

**Table 1:** Pt(II)-Catalyzed synthesis of cyclohexenone from 1,6-diyne *^a^*.

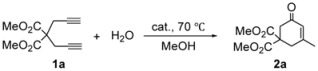				
Entry	Catalyst (mol%)	CH_3_SO_3_H (mol%)	Time (h)	Yield *^b ^* (%)
1	Pt(COD)Cl_2_ (5)	50	3	75
2	Pt(COD)Cl_2_ (5)	-	3	Trace
3	Pt(COD)Cl_2_ (2)	50	3	21
4	Pt(PPh_3_)Cl_2_ (2)	50	4	12
5	Pd(P*^i^*Pr_3_)Cl_2_ (2)	50	4	NR
6	Pt(COD)Cl_2_ (2)	50	4	Nr

^a^ The reactions were performed with 1a (0.5 mmol), H_2_O (0.5 mmol), CH3SO3H (1-50 mol%), and catalyst (2-5 mol%) in MeOH (2 mL) at 70 °C. ^b^ Isolated yields.

In order to demonstrate the efficiency and scope of the present method, we applied the optimum conditions of entry 1 in Table 1 to the hydrative cyclization of several 1,6-diyne substrates bearing a variety of functionalities at their 4-positions. The results are summarized in Table 2. Terminal malonate derivatives **1a** and **1b** were found to be good substrates (Table 2, entries 1 and 2). This is quite similar to the results of Au (I)–catalyzed reactions [24,25]. To our delight, the presence of two hydroxyl groups as in compound **1c** was tolerated, thus providing cyclohexenone **2c** bearing hydroxyl groups with no intramolecular alcohol addition products (Table 2, entry 3) [26,27]. Protecting groups such as the single methyl ether in **1d** or the double methyl ether in **1e** were also compatible with the present method (Table 2, entries 4 and 5). Cyclic products with different substituent group pairs, such as the diphenylphosphoryl and ethoxycarbonyl in **2f**, or the phenyl and methoxycarbonyl in **2h**, were also obtained in good yields (Table 2, entries 6 and 8). The acetylacetone derivative **1i** and its reduced derivative **1j** were transformed into cyclic products **2i** and **2j **(Table 2, entries 9 and 10). In our hands the spirocyclic compound **2k** bearing a fluorene moiety was successfully obtained from diyne **1k** in 48% yield (Table 2, entry 11).

**Table 2:** Pt (II) catalyze hydrative cyclyzation reaction of 1, 6-heptadiynes ^a^.

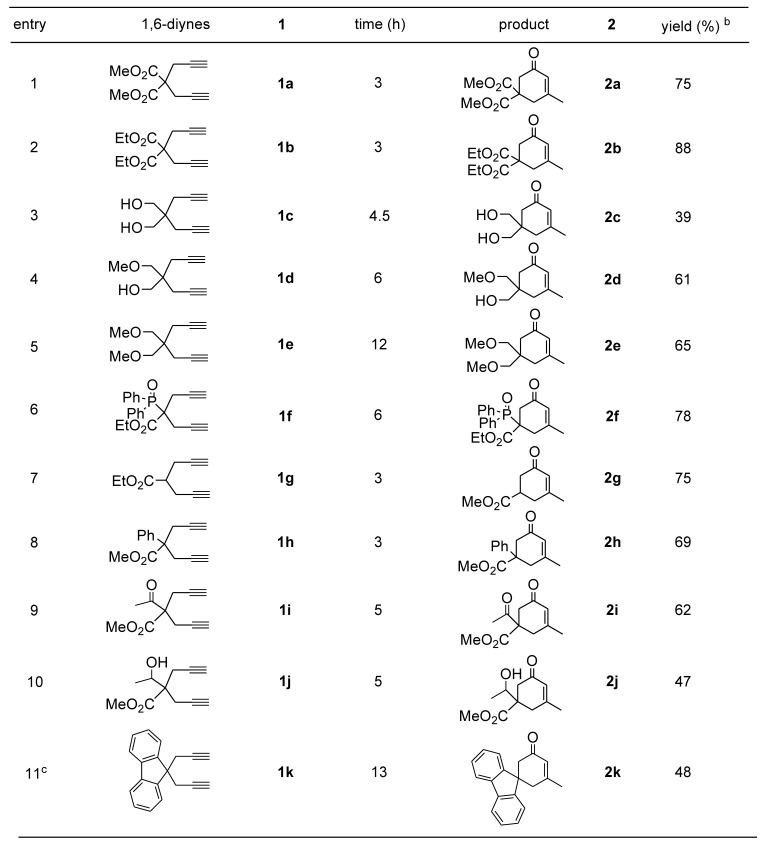

*^a^* All reactions were performed with 0.5 mmol of substrate, 0.5 mmol of H_2_O, 0.25 mmol of CH_3_SO_3_H, and 5 mol% of Pt(COD)Cl_2_ in 2.0 mL of MeOH at 70 °C; *^b^* Isolated yields; *^c^* 1 mL of MeOH and 1 mL of CH_2_Cl_2_ were used as solvent.

**Scheme 1:**
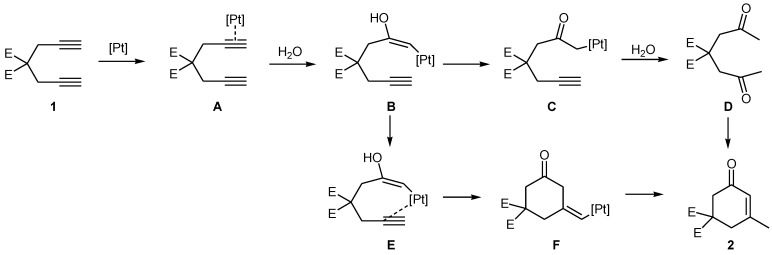
Proposed mechanism for The Pt-catalyzed hydrative cyclization of 1, 6-diynes.

Presumably, the mechanism in this reaction could be similar to that of the PtCl_2_-catalyzed hydrative cyclization of trialkyne functionalities [7]. We thus propose a mechanism (Scheme 1) involving an initial coordination of the diyne to Pt(II) to afford the intermediate **A**. The addition of H_2_O takes place to form the α-carbonyl platinum species **C**. After a second hydration at the remaining alkyne of species **C**, the resulting diketone species **D** undergoes a subsequent aldol condensation to form a product **2**. Alternatively, cyclohexenone 2 could result from an alkyne insertion into intermediate **E**, followed by hydrodemetalation of intermediate **F**. The reason behind the catalytic activity of acid as an additive is unclear, although acid is proposed to exert a tuning effect on the activity of Pt catalysts.

## 3. Experimental

### 3.1. General

Under otherwise noted, materials were obtained from commercial suppliers and used without further purification. Diynes were prepared by the procedures in the literature [29,30]. Thin layer chromatography (TLC) was performed using silica gel 60 F_254_ and visualized using UV light. Column chromatography was performed with silica gel (mesh 300-400). ^1^H-NMR and ^13^C-NMR spectra were recorded on a Bruker Avance 400 MHz or 500 MHz spectrometer in CDCl_3_ with Me_4_Si as an internal standard. Data are reported as follows: chemical shifts in ppm (δ), multiplicity (s = singlet, d = doublet, t = triplet, q = quartet, br = broad and m = multiplet, coupling constant (Hz) and integration. Infrared spectra (IR) were obtained on a 370 FT-IR spectrometer; absorptions are reported in cm^-1^. Mass spectra (MS) and high resolution mass spectra (HRMS) were obtained at the Zhejiang University of Technology Mass Spectrometry Facility.

### 3.2. General procedure for the hydrative cyclization of diynes

To a reactor containing diyne (0.5 mmol), methanol (2 mL), and H_2_O (10 μL) under nitrogen Pt(COD)Cl_2_ (9.0 mg, 0.025 mmol, 5 mol%) and CH_3_SO_3_H (20 μL) were added. The resulting yellow solution was then sealed and stirred at 70 °C for 3-13 hours until the starting diyne was consumed, as judged by TLC. The mixture was quenched with a saturated solution of NaHCO_3_ and then extracted with ethyl acetate (20 mL × 3). The organic layer was washed with brine, dried over Na_2_SO_4_ and concentrated *in vacuo*. The residue was purified by column chromatography (silica gel) (Eluent: hexane/ethyl acetate) to yield the corresponding cyclized product in an analytically pure form.

*Dimethyl 3-methyl-5-oxocyclohex-3-ene-1,1-dicarboxylate (**2a**)* [24]. A pale yellow oil; ^1^H-NMR (400 MHz, CDCl_3_) *δ* 5.88 (s, 1H), 3.75 (s, 6H), 2.90 (s, 2H), 2.87 (s, 2H), 2.01 (s, 3H); ^13^C-NMR (100 MHz, CDCl_3_) *δ* 194.5, 170.2, 158.7, 126.2, 55.5, 53.3, 41.7, 36.3, 24.3.

*Diethyl 3-methyl-5-oxocyclohex-3-ene-1,1-dicarboxylate* (**2b**) [24]. A colorless oil; ^1^H-NMR (400 MHz, CDCl_3_) *δ* 5.88 (q, *J* = 1.2 Hz, 1H), 4.20 (q, *J* = 7.0 Hz, 4H), 2.89 (s, 2H), 2.86 (s, 2H), 2.01 (d, *J* = 1.2 Hz, 3H), 1.24 (t, *J* = 7.0 Hz, 6 H); ^13^C-NMR (100 MHz, CDCl_3_) *δ* 194.8, 169.8, 158.7, 126.2, 62.2, 55.5, 41.7, 36.2, 24.3, 13.9.

*5,5-Bis(hydroxymethyl)-3-methylcyclohex-2-enone* (**2c**) [25]. White solid, m.p.: 64–65 °C. ^1^H-NMR (500 MHz, CDCl_3_) *δ* 5.88 (s, 1H), 3.91 (br, 2H), 3.55 (s, 4H), 2.30 (s, 4H), 1.98 (s, 3H); ^13^C-NMR (125 MHz, CDCl_3_) *δ* 199.8, 161.4, 125.6, 66.5, 42.4, 40.8, 35.0, 24.6.

*5-(Hydroxymethyl)-5-(methoxymethyl)-3-methylcyclohex-2-enone* (**2d**). A pale yellow oil; ^1^H-NMR (400 MHz, CDCl_3_) δ 5.84-5.83 (m, 1H), 3.53-3.45 (m, 2H), 3.35-3.26 (m, 5H), 2.88 (br, 1H), 2.36 (s, 2H), 2.24 (s, 2H) , 1.92 (s, 3H); ^13^C-NMR (100 MHz, CDCl_3_) δ 198.6, 160.2, 125.6, 77.5, 67.2, 59.4, 41.8, 41.2, 34.9, 24.2; IR (KBr) υ_max _3445, 2927, 1651, 1382, 1104 cm^-1^; HRMS (EI) for C_10_H_16_O_3_: calcd. 184.1099. Found 184.1097.

*5,5-Bis(methoxymethyl)-3-methylcyclohex-2-enone* (**2e**) [24]. A pale yellow oil; ^1^H-NMR (400 MHz, CDCl_3_) *δ* 5.86 (s, 1H), 3.31 (s, 6H), 3.23 (s, 4H), 2.34 (s, 2H), 2.32 (s, 2H), 1.94 (s, 3H); ^13^C-NMR (100 MHz, CDCl_3_) *δ* 198.9, 159.8, 125.4, 75.4, 59.2, 41.6, 41.3, 34.8, 24.3.

*Ethyl 1-(diphenylphosphoryl)-3-methyl-5-oxocyclohex-3-enecarboxylate* (**2f**) [24]. White solid, m.p. 122.3–125.5 °C.^ 1^H NMR (400 MHz, CDCl_3_) *δ* 8.06-8.02 (m, 2H), 7.90-7.85 (m, 2H), 7.69-7.47 (m, 6H), 5.84 (s, 1H), 3.93-3.79 (m, 2H), 3.03-2.83 (m, 4H), 1.92 (s, 3H), 0.91-0.86 (m, 3H); ^13^C-NMR (100 MHz, CDCl_3_) *δ* 194.4 (d, *J* c-p = 11.3 Hz), 170.8 (d, *J* c-p = 20.2 Hz), 159.2 (d, *J* c-p = 12.4 Hz), 132.2 (q, *J* c-p = 2.8 Hz), 131.9 (d, *J* c-p = 8.9 Hz), 131.6 (d, *J* c-p = 8.9 Hz), 129.0 (d, *J* c-p = 10.6 Hz), 128.3 (d, *J* c-p = 2 Hz), 128.2 (d, *J* c-p = 2 Hz), 128.0 (d, *J* c-p = 11 Hz), 125.6, 61.7, 53.0 (d, *J* c-p = 57 Hz), 39.1, 33.9, 24.1, 20.6 (d, *J* c-p = 4.1 Hz), 13.1.

*Methyl 3-methyl-5-oxocyclohex-3-enecarboxylate* (**2g**) [24]. A pale yellow oil; ^1^H-NMR (400 MHz, CDCl_3_) *δ* 5.91 (s, 1H), 3.72 (s, 3H), 3.10-3.04 (m, 1H), 2.67-2.51 (m, 4H), 2.00 (s, 3H); ^13^C-NMR (100 MHz, CDCl_3_) *δ* 196.8, 173.5, 160.2, 126.5, 52.1, 39.6, 38.6, 33.0, 24.2.

*Methyl 3-methyl-5-oxo-1-phenylcyclohex-3-enecarboxylate* (**2h**) [24]. Colorless crystals; m.p. 83.0-84.0. ^1^H-NMR (400 MHz, CDCl_3_) *δ* 7.37-7.28 (m, 5H), 5.94-5.93 (m, 1H), 3.64 (s, 3H), 3.29-3.21 (m, 2H), 2.81-2.73 (m, 2H), 2.05 (s, 3H); ^13^C-NMR (100 MHz, CDCl_3_) *δ* 196.6, 174.0, 160.4, 140.0, 128.9, 127.7, 126.5, 125.5, 52.8, 51.8, 45.1, 40.1, 24.6.

*Methyl 1-acetyl-3-methyl-5-oxocyclohex-3-enecarboxylate* (**2i**) [25]. A pale yellow oil; ^1^H NMR (400 MHz, CDCl_3_) *δ* 5.87 (s, 1H), 3.76 (s, 3H), 2.93 (d, *J* = 16.4 Hz, 1H), 2.84 (d, *J* = 0.8 Hz, 2H), 2.72 (d, *J* = 16.4 Hz, 1H), 2.20 (s, 3H), 2.01 (s, 3H);^ 13^C NMR (100 MHz, CDCl_3_) *δ* 202.0, 194. 8, 170.9, 158.8, 126.1, 61.6, 53.2, 41.2, 35.4, 25.8, 24.3.

*Methyl 1-(1-hydroxyethyl)-3-methyl-5-oxocyclohex-3-enecarboxylate* (**2j**) [25]. A pale yellow oil; ^1^H NMR (400 MHz, CDCl_3_) *δ* 5.85 (s, 1H), 3.87-2.84 (m, 1H), 3.69 (s, 3H), 2.88-2.79 (m, 2H), 2.70-2.66 (br, 1H), 2.62-2.36 (m, 2H), 2.16-1.84 (m, 3H), 1.25-1.17 (m, 3H); ^13^C-NMR (100 MHz, CDCl_3_) *δ* 197.5, 174.7, 160.4, 126.0, 71.5, 54.0, 52.4, 41.6, 35.5, 24.5, 18.7.

*5-Fluorene-3-methylcyclohex-2-*enone (**2k**) [25]. A white solid; m.p. 167-168 °C; ^1^H-NMR (500 MHz, CDCl_3_) *δ* 7.74 (d, *J* = 7.5 Hz, 2H), 7.48 (d, *J* = 7.5 Hz, 2H), 7.40-7.37 (m, 2H), 7.29-7.26 (m, 2H), 6.27 (d, *J* = 1 Hz, 1H), 2.68 (s, 4H) , 2.02 (s, 3H); ^13^C-NMR (125 MHz, CDCl_3_) *δ* 198.1, 160.4, 149.9, 139.3, 127.9, 127.6, 127.1, 123.0, 120.1, 51.0, 45.7, 41.2, 24.5.

## 4. Conclusions

In summary, various 3,5-substituted conjugated cyclohexenones were synthesized by Pt(II)–catalyzed hydrative cyclization of 1,6-diynes. Advantages of the present method are the easily accessible starting materials, mild conditions, lack of coproducts and the fact that several types of functional groups were tolerated. Further studies are underway to expand the scope of the present method and are directed toward further method development on these cyclohexenone scaffolds as well as applications in natural product and the bioactive molecule synthesis.
